# A nationwide study on reproductive function, ovarian reserve, and risk of premature menopause in female survivors of childhood cancer: design and methodological challenges

**DOI:** 10.1186/1471-2407-12-363

**Published:** 2012-08-23

**Authors:** Annelies Overbeek, Marleen H van den Berg, Leontien CM Kremer, Marry M van den Heuvel-Eibrink, Wim JE Tissing, Jacqueline J Loonen, Birgitta Versluys, Dorine Bresters, Gertjan JL Kaspers, Cornelis B Lambalk, Flora E van Leeuwen, Eline van Dulmen-den Broeder

**Affiliations:** 1 Department of Paediatrics, Division of Paediatric Oncology/Haematology, VU University Medical Center, PO Box 7057, Amsterdam, 1007MB, The Netherlands; 2 Department of Obstetrics and Gynaecology, VU University Medical Center, Amsterdam, The Netherlands; 3 Department of Paediatric Oncology, Emma Children’s Hospital/Academic Medical Center, Amsterdam, The Netherlands; 4 Department of Pediatric Oncology, Sophia Children’s Hospital/Erasmus MC University Medical Center, Rotterdam, The Netherlands; 5 Department of Pediatric Oncology, Beatrix Children’s Hospital/University Medical Center Groningen, Groningen, The Netherlands; 6 Department of Pediatric Oncology, Radboud University Nijmegen Medical Center, Nijmegen, The Netherlands; 7 Department of Pediatric Oncology, Wilhelmina’s Children’s Hospital, University Medical Center Utrecht, Utrecht, The Netherlands; 8 Department of Pediatric Stem Cell Transplantation, Willem-Alexander Children’s Hospital, Leiden University Medical Center, Leiden, The Netherlands; 9 Department of Epidemiology, Netherlands Cancer Institute, Amsterdam, The Netherlands

**Keywords:** Childhood cancer survivors, Adverse effects, Female fertility, Study design, Methodological challenges

## Abstract

**Background:**

Advances in childhood cancer treatment over the past decades have significantly improved survival, resulting in a rapidly growing group of survivors. However, both chemo- and radiotherapy may adversely affect reproductive function. This paper describes the design and encountered methodological challenges of a nationwide study in the Netherlands investigating the effects of treatment on reproductive function, ovarian reserve, premature menopause and pregnancy outcomes in female childhood cancer survivors (CCS), the DCOG LATER-VEVO study.

**Methods:**

The study is a retrospective cohort study consisting of two parts: a questionnaire assessing medical, menstrual, and obstetric history, and a clinical assessment evaluating ovarian and uterine function by hormonal analyses and transvaginal ultrasound measurements. The eligible study population consists of adult female 5-year survivors of childhood cancer treated in the Netherlands, whereas the control group consists of age-matched sisters of the participating CCS. To date, study invitations have been sent to 1611 CCS and 429 sister controls, of which 1215 (75%) and 333 (78%) have responded so far. Of these responders, the majority consented to participate in both parts of the study (53% vs. 65% for CCS and sister controls respectively). Several challenges were encountered involving the study population: dealing with bias due to the differences in characteristics of several types of (non-) participants and finding an adequately sized and well-matched control group. Moreover, the challenges related to the data collection process included: differences in response rates between web-based and paper-based questionnaires, validity of self-reported outcomes, interpretation of clinical measurements of women using hormonal contraceptives, and inter- and intra-observer variation of the ultrasound measurements.

**Discussion:**

The DCOG LATER-VEVO study will provide valuable information about the reproductive potential of paediatric cancer patients as well as long-term survivors of childhood cancer. Other investigators planning to conduct large cohort studies on late effects may encounter similar challenges as those encountered during this study. The solutions to these challenges described in this paper may be useful to these investigators.

**Trial registration:**

NTR2922; http://www.trialregister.nl/trialreg/admin/rctview.asp?TC=2922

## Background

Cancer treatment can have detrimental effects on reproductive function. In women, there is evidence that both chemo- and radiotherapy can adversely affect ovarian function, ovarian reserve and uterine function, clinically leading to sub- or infertility, premature menopause and/or adverse pregnancy outcomes [1-9]. However, previous studies addressing the late effects of cancer treatment on female fertility have several limitations. Clinical ovarian reserve tests are often lacking (i.e. data from questionnaires only) [1-6,10-19], and study populations are often small and heterogeneous [20-27]. Therefore, we designed the DCOG LATER-VEVO study (Dutch Childhood Oncology Group - Long term Effects after Childhood Cancer/ Fertility, Ovarian reserve and Premature Menopause (Dutch acronym)) in the Netherlands. Patient inclusion started in 2008.

The study aims to evaluate the effects of cancer treatment on the reproductive system of female childhood cancer survivors (CCS) in the Netherlands and their risk of premature menopause. The effects of treatment in general will be assessed, as well as the effects of different treatment modalities, doses of drugs, radiation sites and doses, and age at time of treatment. The study includes a questionnaire survey and a full panel of ovarian function and reserve tests. The DCOG LATER-VEVO study is the first nationwide childhood cancer survivor study in the Netherlands and during the study period several methodological challenges were encountered.

In this paper the key methodological and practical challenges are discussed as well as the way they were addressed. Other investigators planning to conduct large nationwide cohort studies among childhood cancer survivors will benefit from this information when faced with similar challenges.

## Patients and methods

### Design and study population

The DCOG LATER-VEVO study is a multi-center retrospective cohort study including female 5-year survivors of childhood cancer. The study consists of three parts: a questionnaire survey, the provision of a blood sample, and a transvaginal ultrasound measurement of the reproductive organs, the latter two requiring a visit to the outpatient clinic. Approval was obtained from the relevant medical ethics committees and written informed consent was obtained from all participants.

Eligible cohort members are selected from a cohort of patients treated for childhood cancer between 1963 and 2002 at one of the seven Dutch paediatric oncology - and stem cell transplant centers, collectively known as the Dutch Childhood Oncology Group - Longterm Effects after Childhood Cancer (DCOG LATER). This group has developed a nationwide electronic database including patient and treatment details of all CCS in the Netherlands (DCOG LATER database). The study population consists of those female CCS who were treated for a malignancy or central nervous system tumour before the age of 18, who survived for at least 5 years after diagnosis, and who were at least 18 years at study entry (n = 2331). The exclusion criteria for participation in the study include: deceased before the start of the study (n = 271), living abroad or unknown address (n = 75), not being able to speak or read Dutch (n = 1), having severe mental sequelae (n = 40), being treated for second malignant neoplasm at the time of study inclusion (n = 34), and previously having indicated not willing to participate in research (n = 13). Thus, a total of 1897 female childhood cancer survivors are eligible for participation in the DCOG LATER-VEVO study.

Sisters of participating CCS who have never been diagnosed with cancer, who are able to read and speak Dutch, and who are 18 years or older, are asked to participate in the control group of the study. For this purpose participating CCS are asked to contact all sisters meeting the inclusion criteria and to provide their contact information to the investigators. If a female survivor chooses to not register one or more available sisters, the reason is enquired about.

### Approach of study participants and data collection

**Figure 1:**
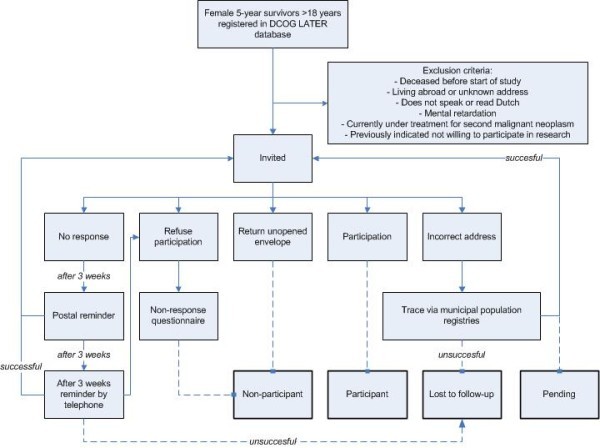
**Response flow chart.** The following categories and definitions are used to classify participants and non-participants in the DCOG LATER-VEVO study: 1) “eligible subjects” are individuals registered in the DCOG LATER database who were confirmed as meeting the study eligibility criteria; 2) “participants” are those who consented to participate; 3) “non-participants” are individuals who declined participation verbally or in writing, who returned the envelope with study information unopened or who, at first indicated they were willing to participate but ultimately did not do so; 4) subjects are considered “lost to follow-up” if they were not located after intensive tracing efforts; and 5) individuals are classified as “pending” when they are actively being traced and recruited to participate.

All eligible women receive a mailed package containing extensive study information, an informed consent and refusal form, and a questionnaire. They are asked to complete the questionnaire and return it with a signed informed consent form. Furthermore, they are asked to indicate on the informed consent form in which parts of the study they are willing to participate. In case of no response within 3 weeks, postal reminders are sent. When again after 3 weeks no response has been received, the women are contacted by telephone. Women who are not willing to participate in either part of the study are asked to complete a refusal form on which they can indicate the reason for not wanting to participate. These non-participants are asked to complete a brief questionnaire regarding parity, wish to have children, subfertility, subfertility treatment, and educational level in order to adjust for possible bias. The envelope containing the study information package is sealed and put in another envelop, together with a cover letter in which the study is explained very briefly. This is done in order to give the survivors the opportunity to choose whether or not they want to be confronted with the extensive study information. If not, they can send the unopened package return to sender. Figure 1 depicts the various response categories that apply to the DCOG LATER-VEVO study.

The data collected for survivors and siblings are the same, with the exception of data related to the anti-cancer treatment in the past. For both survivors and siblings information on reproductive and medical history is obtained by a questionnaire which is available either as hard copy or online. This questionnaire is an adaptation of a well-tested questionnaire used by the Department of Epidemiology of the Netherlands Cancer Institute in a large-scale Dutch cohort study of long-term effects of ovarian stimulation for in vitro fertilization [28,29]. It addresses the following issues: socio-demographic characteristics, menstrual history, desire to have children, reproductive history, pregnancy information, pregnancy outcomes, details of offspring, menopausal symptoms and menopause, use and duration of use of exogenous hormones, use and duration of use of fertility drugs and assisted reproductive techniques, family history of cancer and sub-/infertility, co-morbidities, and life style behaviour.

In order to assess reproductive function and ovarian reserve a blood sample is drawn and a transvaginal ultrasound of the reproductive organs is performed. From the blood sample, FSH, LH, estradiol, inhibin-B, prolactin, and AMH concentrations are determined as well as the FSH receptor genotype. The ultrasound measurements, which assess the number of antral follicles in both ovaries as well as the length and width of the ovaries and the uterus, are performed by specifically trained personnel using a HD11 XE ultrasound system with a transvaginal probe which can perform three-dimensional (3D) imaging (EnVisor HD, Philips Medical Systems, Eindhoven, the Netherlands). First, a 2D ultrasound assessment of the pelvis is performed after which an automated mechanical sweep produces the 3D data. An ultrasound measurement is not performed when the participant indicates in the questionnaire that she has not yet been involved in sexual intercourse, unless she explicitly states she wants to undergo an ultrasound. Both the blood sampling and ultrasound measurements require specific timing. For both CCS and controls not using hormonal contraceptives this timing is as follows: (1) on day 2–5 of a natural menstrual cycle; (2) on any convenient moment in case of amenorrhea (no menses > 6 months). In those who are using hormonal contraceptives, alternative methods of timing were used (see section “The value of hormonal and ultrasound markers while using oral contraceptives”).

Since January 2008, invitations have been sent to 1611 female CCS and 429 sister controls from all participating centers and data collection is still ongoing. As of March 1^st^ 2012, 1215 CCS and 333 sister controls have responded, whereas from the remaining 396 survivors and 96 controls no response has been received to date. Table 1 describes the response and participation rates of CCS and controls as of March 1^st^ 2012.

**Table 1:** Response and participation rates of childhood cancer survivors and sister controls in the DCOG LATER-VEVO study*

	**Survivors**	**sister controls**
**Invited**	1611	429
Response received (responders)	1215 (75%)	333 (78%)
No response received (non-responders)	396 (25%)	96 (22%)
**Responders**		
**Participants**		
Questionnaire only	306 (19%)	96 (22%)
Questionnaire and blood sample	126 (8%)	43 (10%)
Questionnaire, blood sample and transvaginal ultrasound	509 (32%)	174 (41%)
**Non-participants**	274 (17%)	20 (5%)

* Rates acquired as of March 1^*st*^*2012*.

## Challenges

The challenges encountered during the study can be divided into two categories: challenges related to the study population and challenges related to the data collection procedures.

### Study population

#### Bias due to different characteristics of the women in the various response groups

Eligible study subjects can either respond (responders) or not respond to the study invitation (non-responders). The responders either decide to participate (participants) or not to participate in the study (non-participants). When they decide to participate they can subsequently choose to take part in one, two or all three parts of the study. In total, three groups of participants are distinguished: 1. questionnaire only; 2. questionnaire and blood sample; 3. questionnaire, blood sample and transvaginal ultrasound. The responders may not be comparable to the non-responders and the same is true for non-participants versus participants. In addition, subjects who choose to complete the questionnaire only may not be comparable to those who also participate in the clinical assessment. This may potentially lead to selection bias, which may influence the validity of the study results. Therefore, it is important to identify potential differences between the various response groups in order to be able to control for selection bias during the data analyses of the DCOG LATER-VEVO study.

In order to identify the presence and direction of possible selection bias interim data analyses were performed, in which differences between the characteristics of responders and non-responders were compared. Results showed that the age at study invitation (28.8 vs. 28.2 years), age at diagnosis (6.96 vs. 7.06 years) and time since treatment (7998 vs. 7658 days) were not different between responders and non-responders. In addition, differences between the characteristics of participants and non-participants were compared. Results showed that the non-participating CCS (n = 274) did not significantly differ from the participating CCS regarding current age (p = 0.09). In addition, there were no significant differences in age at diagnosis or in time since diagnosis (p = 0.23 and p = 0.24, respectively). Of the 274 non-participants, 17% (n = 46) were willing to complete the brief non-participant questionnaire. Data from this questionnaire showed no significant differences with regard to educational level, although the proportion of women with a high educational level in the participants group was substantially higher compared to the non-participants group (39.9% vs. 26.1%, p = 0.09). Moreover, a larger proportion of the non-participants reported to already have offspring in comparison with participants. However, this difference was not significant either (46.3% vs. 33.9%, p = 0.10). Nevertheless, this may suggest that women with proven fertility may be less likely to participate in the study than those who have not given birth (yet). This implies that caution should be taken when interpreting the results of the DCOG LATER-VEVO study since an overestimation of the adverse effects of the cancer treatment on reproductive outcomes (i.e. actual fertility) might be introduced. We realize, however, that the number of women completing the non-participant questionnaire was rather low. As a consequence, this group of women may not be fully representative of the total group of non-participants. However, the non-participants that did and those who did not complete the non-participant questionnaire appeared not to significantly differ regarding current age, age at diagnosis, and time since diagnosis.

With regard to possible selection bias within the participating groups, we evaluated the differences in characteristics between those participating in the questionnaire part only and those participating in both the questionnaire and the clinical part (blood and/or ultrasound) of the study (Table 2). It appeared that women who completed the questionnaire only were older, had a longer follow-up time since diagnosis, were less likely to be highly educated, were more likely to have had intercourse and to have offspring than women who also agreed to participate in the clinical part.

**Table 2:** Comparison of several characteristics between two different groups of participants

**Variable**	**Participants**		**P value**
	**Participating in questionnaire part only (n = 306)**	**Participating in questionnaire and clinical part (n = 635)**	
**Age (years)**	30 (18–52)	27 (18–56)	<0.001
**Age at diagnosis (years)**	6 (0–16)	6 (0–17)	NS
**Time since diagnosis (days)**	8280.5 (2563–15423)	7418 (2068–14612)	0.001
High education (n/N) (%)*	103/300 (34.3)	267/619 (43.1)	0.002
In committed relationship (n/N) (%)*	217/304 (71.4)	431/633 (68.1)	NS
Has had intercourse (n/N) (%)*	248/303 (81.8)	555/631 (88.0)	0.01
Has offspring (n/N) (%)*	146/304 (48.0)	183/633 (28.9)	<0.001
Has previously consulted a gynaecologist for fertility problems (n/N) (%)*	46/289 (15.9)	72/621 (11.6)	NS

Data presented as median (range) unless indicated otherwise. NS = not significant. * Total N does not correspond with the number mentioned in the heading of the table because of missing data.

#### Participants who ultimately did not show up for outpatient clinic visit

If participants decide to provide a blood sample and/or undergo a transvaginal ultrasound, they are asked to contact the research staff on a specific day of their menstrual cycle in order to plan the clinical assessment at the outpatient clinic. However, we experienced that some participants who initially consented to a clinical assessment did not (yet) follow-up on their decision 6 months after their written consent (n = 90) . Twenty-seven women (30%) have expressed a reason (pregnancy, breastfeeding, illness or disability, and personal or family responsibilities) while the remaining 63 have not yet contacted the research staff, even after several reminders. Ultimately, these women will be classified as non-participants to the clinical part of the study.

#### Finding an adequately sized and well-matched control group

Sisters of participating CCS are invited as controls, since they have the same genetic and socio-economic background (which might influence fertility and other outcomes). However, the inclusion of only sisters in the control group has shown to result in insufficient numbers compared to the number of CCS (941 versus 313, see also Table 1). The reason is two-fold: not all participating CCS have an eligible sister aged 18 years or older and not all CCS with eligible sisters gave permission to contact their sisters for the control group. For the DCOG LATER-VEVO study this may ultimately result in insufficient power for certain subgroup analyses. Moreover, not all research questions of the study require a control group that is genetically and/or socioeconomically comparable to the survivor group. Therefore, it was decided to expand the control group by including women from the general population as well.

For this purpose, general practitioners of the participating CCS are asked to randomly select and invite subjects from their female patient population. For logistical reasons, we selected general practitioners practices located in the area surrounding the coordinating center. In order to ensure a comparable age distribution between CCS and controls, these general practitioners are asked to select women within a specified age range of five years (so-called “GP controls”). For these women, the same inclusion criteria apply as for the sister controls: never to have been diagnosed with cancer, able to read and speak Dutch, and 18 years or older. This method of approach resulted in 1184 women who were invited to participate in the DCOG LATER-VEVO study as controls. So far, 935 (79%) have responded and 429 women have consented to participate, 308 of whom in the clinical part of the study.

Within the group of eligible controls recruited via the general practitioners, we conducted a non-responder analysis. A random sample (n = 200) was drawn from the GP controls who did not respond to the study invitation. From these women the following variables were collected from the medical records at the general practitioner’s office: age, having offspring, maternal age at first childbirth, fertility-related problems, and visits to gynaecologists. These data, which were made anonymous for privacy reasons, were compared with data of a random sample of GP controls who did respond to the study invitation (n = 194). Preliminary results show no significant differences between the two groups with respect to the before-mentioned variables. This suggests that the degree of selective participation within the GP control group is low.

We also evaluated whether sister controls differed from the GP controls on several basic characteristics (Table 3). Results show that GP controls were more likely to have a high educational level. In addition, these women were older than sister controls. There were no significant differences between siblings and GP controls with regard to relationships, offspring or fertility issues.

**Table 3:** Differences between sister controls and controls recruited through the general practitioner (GP controls)

	**sister controls (n = 313)**	**GP controls (n = 430)**	**P value**
**Age**	30 (18–58)	34 (18–54)	<0.001
**High education (n/N) (%)**	158/308 (51.3)	293/428 (68.5)	<0.001
**Is in committed relationship (n/N) (%)**	249/311 (80.1)	341/428 (79.7)	NS
**Has had intercourse (n/N) (%)**	288/312 (92.3)	407/426 (95.5)	NS
**Has offspring (n/N) (%)**	140/313 (44.7)	201/429 (46.9)	NS
**Has previously consulted a gynaecologist for fertility problems (n/N) (%)**	29/301 (9.6)	44/411 (10.7)	NS

*Data presented as median (range) unless indicated otherwise. NS = not significant*.

### Data collection

#### Differences in response between web-based and paper-based questionnaires

Web-based questionnaires have several advantages over paper-based questionnaires. They are less time-consuming, less costly, and the data of the respondents are already available in an electronic format, leading to less input errors [30-32]. At the time the DCOG LATER-VEVO study was set up, no literature was available concerning the differences in response rates of CCS to a web-based or a hard copy paper questionnaire, when they are offered both types of questionnaire. Therefore, we conducted a nested randomized study to evaluate whether the use of either the web-based or hard copy paper questionnaire resulted in differences in response rates, type of response, and characteristics of the (non-)responders. In this study, 277 eligible women were randomly selected to receive either a mixed invitation (a hard copy paper questionnaire together with the login details for the web-based questionnaire) or a web-only invitation (login details only). Women receiving the web-only invitation were given the opportunity to apply for a hard copy of the questionnaire by returning a form. The results showed that although the overall response rates to both types of invitation were similar, adding a paper version of a questionnaire to a web-only invitation resulted in more respondents completing the hard copy of the questionnaire. In addition, women who were older, higher educated as well as those who were a student, had a higher probability of completing the web-based version of the questionnaire (Table 4) [33]. It was decided that future invitations for the DCOG LATER-VEVO study should include both a hard copy of the questionnaire and the login details for completing the web-based questionnaire.

**Table 4:** Factors associated with the probability of completing the web-based version of the questionnaire: results of logistic regression*

	**OR (95% CI)**
**Age**	1.08 (1.02-1.15)
**Educational level (ref. group: High level)**	
** Medium**	0.65 (0.28-1.53)
** Low**	0.06 (0.01-0.52)
**Employment status (ref. group: Employed)**	
** Student**	3.25 (1.00-10.56)
** Unemployed**	0.35 (0.10-1.29)
**Randomization group (ref. group: Mixed invitation group)**	2.85 (1.31-6.21)

*OR, odds ratio; CI, confidence interval*.

* Derived from Van den Berg MH et al. Using web-based and paper-based questionnaires for collecting data on fertility issues among female cancer survivors: differences in response characteristics. J Med Internet Res. 2011 Sep 29;13(3):e76.

#### Validity of self-reported outcomes

Previous studies on fertility and pregnancy outcomes in CCS were often designed as large cohort studies in which the outcomes of interest were obtained through interviews or mailed questionnaires [4,13]. However, the reliability and validity of self-reported data can be limited and one should be aware of possible (non-) differential misclassification bias. Several studies among healthy women have been performed assessing the accuracy and validity of self-report for pregnancy outcomes. Data from these studies showed that birth weight and gestational age were accurately reported, but time to pregnancy, reasons for subfertility, and maternal and neonatal complications during labour and delivery were reported with less accuracy [34-36]. Since no literature was available assessing the validity of self-reported pregnancy outcomes by CCS, we conducted a validation study among our study participants [37]. Women were eligible for the validation study when they reported in the questionnaire to have had a child between 1/1/1985 and 31/12/2009. Reference data on pregnancies and pregnancy outcomes were abstracted from the Netherlands Perinatal Registry (PRN). In this nationwide population-based registry pregnancy outcomes of all births are registered by midwives, obstetricians and paediatricians and data are available from 1985 to 2009. Records of self-reported pregnancies were linked to the PRN by using both the mother’s date of birth and the child’s date of birth as linkage keys. At the time of the validation study, 879 CCS and 287 controls had returned the study questionnaire. In total, 589 pregnancies were reported in 289 CCS compared to 293 pregnancies in 123 controls. Linkage to the PRN yielded 510 unique hits (345 pregnancies in 186 CCS, and 186 pregnancies in 87 controls). A high intra-class correlation coefficient (ICC) was found for birth weight (0.94 (95%CI 0.91-0.96) and 0.87 (95%CI 0.83-0.90) for CCS and controls, respectively). For gestational age, the ICC was 0.88 for CCS (95%CI 0.85-0.91), but only 0.49 for controls (95%CI 0.32-0.62). The kappa value for method of conception was moderate to good, but varied largely per method (0.56 for hormonal stimulation to 1.0 for IUI). The kappa values for different methods of delivery were good for CCS and controls (0.76 for spontaneous delivery to 0.92 for vacuum/forcipal extraction). Kappa for pregnancy-induced hypertension was 0.59 for CCS and 0.61 for controls. Multilevel analyses showed no differences in accuracy associated with time since pregnancy or educational level.

#### The value of hormonal and ultrasound markers while using oral contraceptives

The results of a pilot study conducted before the start of the DCOG LATER-VEVO study showed that 55% of the CCS used oral contraceptives. As it was anticipated that the response rates to the study would be significantly lower when the participants had to stop using oral contraceptives to be eligible for our study, we conducted a study to compare both hormonal and ultrasound markers of ovarian reserve measured on day 7 of the pill free interval and two subsequent natural cycles. Results showed that FSH and inhibin B values decreased significantly when contraception use was discontinued, whereas values of AMH, AFC and ovarian volume increased significantly. Thus, hormonal and ultrasound markers of ovarian function in oral contraceptive users measured at the end of the hormone-free interval do not fully represent subsequent natural early follicular phase values. However, FSH, AMH and AFC can be used to predict early follicular phase values using calculated prediction equations [38].

The results of this study have led to the following procedures regarding the timing of the clinical measurements of the DCOG LATER-VEVO study. Women who are on oral contraceptives or who use a combined contraceptive vaginal ring are asked to refrain from using these and use other methods of birth control (condoms were provided free of charge), for at least two months prior to the outpatient clinic visit. Depending on the menstrual pattern they develop, these participants are invited according to the timing schedule described above (see section “Approach of study participants and data collection”) during the second natural menstrual cycle. Women who do not wish to discontinue the use of oral contraceptives or vaginal ring during the study period are invited on day 7 of the pill-free or ring-free period for the clinical measurements. Women who use a hormone-containing intrauterine device (IUD) are asked to monitor their basal body temperature (BBT) daily for at least 4 weeks. The BBT chart is evaluated by an experienced gynaecologist (CBL) to detect ovulation. Ovulation is confirmed when the chart appeared to be biphasic (temperature shift of at least 0.5 °C). The date of the assessment for the study is then planned in the early-follicular phase. A monophasic BBT chart was deemed anovulatory and the measurements are planned at any convenient moment. Women using long-acting contraceptive injections or women with a contraceptive implant are excluded from the clinical part of the study.

#### Blinding and inter- and intra-observer variation of ultrasound measurements

To assess ovarian function and ovarian reserve all study participants who consent to this procedure undergo a transvaginal ultrasound. This measurement is performed in five centers across the Netherlands, making this examination as convenient and as timesaving for the participant as possible. All centers are equipped with the same type of 3D ultrasound apparatus. It is not possible to blind ultrasonographers to the CCS status of the participants given the fact that the participants themselves are evidently not blinded to their status and often ask questions regarding their prior cancer treatment during the clinical visit. However, the ultrasonographers do not have access to diagnostic or treatment data prior to or during the procedure. Moreover, the stored 3D files are made anonymous and therefore, no prior knowledge regarding the diagnosis or treatment is available to the investigator analysing the 3D data. This will minimize observer bias.

3D ultrasound is capable of visualizing all three orthogonal planes simultaneously. With the stored volumetric data, imaging can be accurately evaluated offline. Literature shows that both 2D and 3D ultrasound measurements have high intra-observer and inter-observer reliability [38-41], but some limitations were found in the between-method reliability and the degree of agreement when higher numbers of follicles were counted [42]. However, these validation studies have all been performed in healthy controls, or in women undergoing IVF/ICSI treatment. The 2D and 3D techniques have not been validated for women treated for childhood cancer in the past. Former treatment might have induced changes to the reproductive organs, which might result in changes in the intra- and inter-observer reliability of both methods. Moreover, the between-method reliability of real-time 2D images and stored 3D images acquired from CCS has not been investigated so far. Therefore, we are conducting an evaluation of the intra-observer, the inter-observer, and the between-method reliability of both the 2D and 3D ultrasound measurements of the DCOG LATER-VEVO study.

## Discussion

This study on reproductive function, ovarian reserve, and risk of premature menopause in female childhood cancer survivors is the first large nationwide late effects study in the Netherlands. Compared to previously conducted studies on reproductive outcomes in CCS, this DCOG LATER-VEVO study has several strengths.

First, the results of the study are not solely based on self-reported data from questionnaires. Clinical data on ovarian and uterine function are included as well, which allow for a more objective evaluation of the actual fertility status. The extensive set of data acquired in this study will result in detailed knowledge regarding treatment-induced effects on the female reproductive potential, particularly the effects of different types of treatment, doses of drugs, radiation sites and doses, and age at time of treatment.

Second, when data collection of the study is finalized, the reproductive data of the CCS can be compared with those of a large number of controls. For these controls questionnaire as well as clinical data are available. The size of the control group as well as the availability of clinical data from these controls can be considered unique study features within the field of late effect studies among CCS.

Third, the complete cohort of adult female childhood cancer survivors treated in one of seven Dutch paediatric oncology centers is invited to participate in the study, thereby minimizing the risk of selection bias due to loss to follow-up. This is possible because recently a database containing up-to-date patient and treatment data of all Dutch 5-year CCS diagnosed before 2002 has been established. In addition, through the Dutch system of municipal population registries, which fully cover the Dutch population, individuals can nearly always be traced, despite frequent moving. Furthermore, inclusion of the complete cohort provides the advantage of a well-powered study in which several subgroup analyses can reliably be performed.

Despite the above-mentioned strengths of the DCOG LATER-VEVO study, we have also identified several challenges in the design and conduct of the study. To address these challenges, several recommendations have been formulated (Table 5).

**Table 5:** Challenges and recommendations

	**Challenge**	**Recommendations**
**Study population**	Dealing with participation bias	• Keep non-response or loss to follow-up to a minimum
	• Responders and non-responders	• Characterize non-responders or those lost to follow-up
	• articipants and non-participants	• Control for extent and direction of bias in final data analysis
	• Different types of participants	
	• Participants lost to follow-up for the clinical assessment Finding an adequately sized and well-matched control group	• In case the number of controls is insufficient: incorporate other types of control subjects
		• Choose types of controls that are representative of the study population
		• Characterize and control for differences between survivors and controls
		• Compare self-reported data with an more objective source, such as medical records or registries
**Data collection**	Validating instruments for data collection	• Conduct reliability studies to account for inter- and intra- observer variation
		• If possible, use data collection instruments that allow for one investigator to analyse collected data (observer bias)

Our results have shown that there are no significant differences between participants and non-participants regarding socio-demographic data. Nevertheless, a trend was seen towards more highly educated women in the participant group, as well as lower offspring rates. Differences between participants and non-participants may introduce participation bias. This type of bias can be minimized when the number of subjects refusing to participate is kept to a minimum. Moreover, it is important to ask women who do not wish to participate to complete a brief non-participants questionnaire in order to be able to characterize this group. By the time data collection of the DCOG LATER-VEVO study is completed, characteristics of participants and non-participants will be compared once again. By doing so, the extent and direction of participation bias can be established, which can be taken into account when analyzing the data.

During the study period it became clear that a group of participants, who initially consented to visit the outpatient clinic for blood sampling and/or ultrasound measurement, ultimately did not do so. We are making every effort to keep the number of these ‘no-shows’ as low as possible since this selective non-participation might add to selection bias. Indeed, those who feel less inclined to visit the clinic might differ from those who do [43]. At this time, the direction and the magnitude of this bias is difficult to predict. It might be the case that those who ultimately do not visit the clinic may be more fertile and do not see the need for the clinical measurements (anymore). However, these women might also have obtained information that they are infertile. It is therefore of paramount importance that basic information regarding the main outcomes of this study is also collected for the women who ultimately did not show up for the clinical visit. Fortunately, many of these women are seen periodically in outpatient clinics throughout the country for late effects follow-up screening. This enables us to retrieve information regarding reproductive outcomes of these women and to further investigate possible bias.

Two types of controls are included in the study, i.e. sisters of participating CCS and women from the general population recruited through general practitioners. For our study, siblings form a better control group than a random sample from the population, based on a municipal registry sample, neighbourhood controls, or friends and relatives. They share the same genetic and socio-economic background as the CCS. Furthermore, siblings may be more motivated to participate for altruistic reasons, i.e. participating in the study for the sake of their sister, whereas controls from a random sample in the population might have other reasons to participate, for example, fertility problems. This could also lead to selection bias, which could consequently influence our study results. The strong recommendation to register all sisters, and not to choose only one, may further reduce this form of sampling bias.

However, when including only sisters as controls, the number of controls would be significantly lower than the number of survivors. In order to attain sufficient power for the planned statistical analyses, we chose to also include women from the general population. By using a ‘targeted’ invitation strategy we aimed to minimize the risk of selection bias caused by selective participation of, for example, women experiencing fertility problems. Several general practitioners were asked to randomly select and invite women with a specific year of birth from their patient population. In this way, bias due to selective participation is expected to be less compared to applying a broader invitation strategy, for example by using advertisements in daily newspapers to recruit controls. However, it is still probable that some form of selection bias has occurred, since comparisons between the sister controls and GP controls show that the latter group is older and more likely to have a higher educational level.

In order to minimize the travelling time associated with attending the outpatient clinic, only general practitioners located close to the coordinating center (VU University Medical Center Amsterdam) were approached for the recruitment of controls. We realize that this method may introduce additional bias, because of regional differences, which can ultimately lead to selective participation.

Another limitation of the study is the fact that CCS and controls may differ regarding their ability to accurately recall information about past (medical) events, leading to so-called recall or misclassification bias. CCS might better recall their past medical history, because they are frequently seen in outpatient clinics for follow-up, whereas controls may have forgotten about less severe medical problems they have encountered in the past. This might lead to an overestimation of the risk of medical problems in the survivor group. Furthermore, most CCS are aware of the fact that their previous cancer treatment may have had detrimental effects on their fertility. As a consequence, they may report more accurately on several fertility-related items since they have been (or still are) more ‘focused’ on fertility-related events from the past. There is no method available to correct for this type of bias. Nevertheless, the use of objective measurements, such as hormonal analyses and ultrasound examinations, and the validation of self-reported data by comparing with medical records or available registries may minimize the influence of this type of bias.

In order to evaluate the validity of the several outcomes in the study questionnaire, we conducted a study in which self-reported data on pregnancies were compared to data from a nationwide registry. Overall, self-reported pregnancy outcomes of CCS indeed appeared to agree better with the registry data than those of controls. This might be due to the increased awareness of late effects and a higher frequency of medical follow-up. Although self-reported data regarding fertility and pregnancy by CCS seem consistent with registry parameters, differential misclassification between CCS and controls may occur and should be taken into account when interpreting the data. In the near future, we also aim to validate data on fertility issues and gynaecological disorders by comparing the questionnaire data to the medical records.

Clinical measurements for the study were performed at several locations throughout the Netherlands. In order to rule out observer bias, all ultrasound data will be analysed by one investigator using stored 3D ultrasound data. In addition, all endocrinological measurements will be done in a single laboratory at the end of the study, as it is not unlikely that changes in laboratory kits will occur during the study period.

In conclusion, the results of this nationwide study will provide valuable information for counselling female childhood cancer patients and survivors regarding their reproductive potential now and in the future. Other investigators planning to conduct large nationwide cohort studies on late effects may encounter similar challenges as those encountered during this study. Our experiences as well as the way we addressed these challenges will hopefully contribute to an optimal design and conduct of future late effects studies.

## Competing interests

The authors declare that they have no conflict of interest.

## Authors’ contributions

Both AO and MHB wrote the manuscript and contributed equally. All authors contributed to the design of the study and its coordination. AO and MHB are responsible for study logistics, patient recruitment, and data collection. EDB, CBL, FEL, and GJK are principal investigators. EVD was responsible for writing the grant proposals for funding of the study. LCK, MMHE, WJT, JJL, BV, and DB supported local patient recruitment and are clinically responsible for, respectively, the Emma Children’s Hospital/Academic Medical Center, the Sophia Children’s Hospital/Erasmus MC University Medical Center, the Beatrix Children’s Hospital/University Medical Center Groningen, the Radboud University Nijmegen Medical Center, the Wilhelmina’s Children’s Hospital/ University Medical Center Utrecht, the Willem-Alexander Children’s Hospital/Leiden University Medical Center. All authors contributed to and approved the final manuscript.

## Pre-publication history

The pre-publication history for this paper can be accessed here:

http://www.biomedcentral.com/1471-2407/12/363/prepub
